# Supplementary material for: Molecular Crowding Facilitates Ribozyme-Catalyzed RNA Assembly
Source: ACS Cent Sci. 2023 Aug 3;9(8):1670–8. doi: 10.1021/acscentsci.3c00547 (PMC10451029; doi:10.1021/acscentsci.3c00547)
Supplement: Supplementary file 1 — oc3c00547_si_001.pdf [file oc3c00547_si_001.pdf]

Supplementary Information

Molecular Crowding Facilitates Ribozyme-Catalyzed RNA Assembly

Saurja DasGupta[a,b,c,*], Stephanie Zhang[a,d,e], and Jack W. Szostak[a,b,c,d,f,#]

[a] Department of Molecular Biology, Center for Computational and Integrative Biology, Massachusetts General Hospital, Boston, MA 02114, USA.

[b] Howard Hughes Medical Institute, Massachusetts General Hospital, Boston, MA 02114, USA.

[c] Department of Genetics, Harvard Medical School, Boston, MA 02115, USA.

[d] Department of Chemistry and Chemical Biology, Harvard University, Cambridge, MA 02138, USA.

[e] Present address: Department of Pathology, Brigham and Women's Hospital, Boston, Massachusetts 02115, USA

[f] Present address: Howard Hughes Medical Institute, Department of Chemistry, University of Chicago, Chicago, Illinois 60637, USA

[*] Email: dasgupta@molbio.mgh.harvard.edu

[#] Email: jwszostak@uchicago.edu

This PDF file includes:

Table S1

Figures S1 to S10

Name	Sequence (5′→3′)
Ligase 1	GACUCACUGACACAGAUCCACUCACGGACAGCGAAGCUCUCGCCAGCAAAAGAACAGACCGUCGAGGAAACGGCGCUGUCCUUUUUUGGCUAAG**G**
Ligase 2	GACUCACUGACACAGAUCCACUCACGGACAGCGGAAUGCUGCCAACCGUGCGGGCUAAUUGGCAGACUGAGCUCGCUGUCCUUUUUUGGCUAAG**G**
Ligase 3	GACUCACUGACACAGAUCCACUCACGGACAGCGAGCCACUGCGGAAGACCUUAAGAGGUGUAAUUGCUCACCCCGCUGUCCUUUUUUGGCUAAG**G**
Ligation primer	GGCUAAG**G**
Ligation template	GCGGUGGUCCUUAGCC
2AI-activated substrate	(5′-phosphoro-2AI)-ACCACCGCAUUCCGCA
38-6 polymerase	G*GUCAUUG*CCGCACAAAGACAAAUCUCCCCUCAGAGCUUGAGAACAUCUACGGAUGCAGAGGAGGGGCCUUCGGUGGAUCAAUUGUGCACCACCGUUCUCAACACGUACCCGAACAUAAAAAGACCUGACAAAAAGGCGAUGUUAGACACGCACAGGUGCCAUACCCAACACAUGGCUGAC
Polymerization primer	CACUCCACA**C**
Polymerization template	GAC*AAUGAC*AAAAAAAUCAGUACGUCGUGUGGAGUG

Table S1. RNA oligonucleotides used in this study. Fixed base-paired stem nucleotides are underlined. Primer sequences are shown in red and the nucleotide containing the 3′ OH nucleophile is boldfaced. Template sequences complementary to primers are shown in orange. Sequences in 38-6 polymerase and the polymerization template that are complementary and contribute to ribozyme-template binding are italicized.

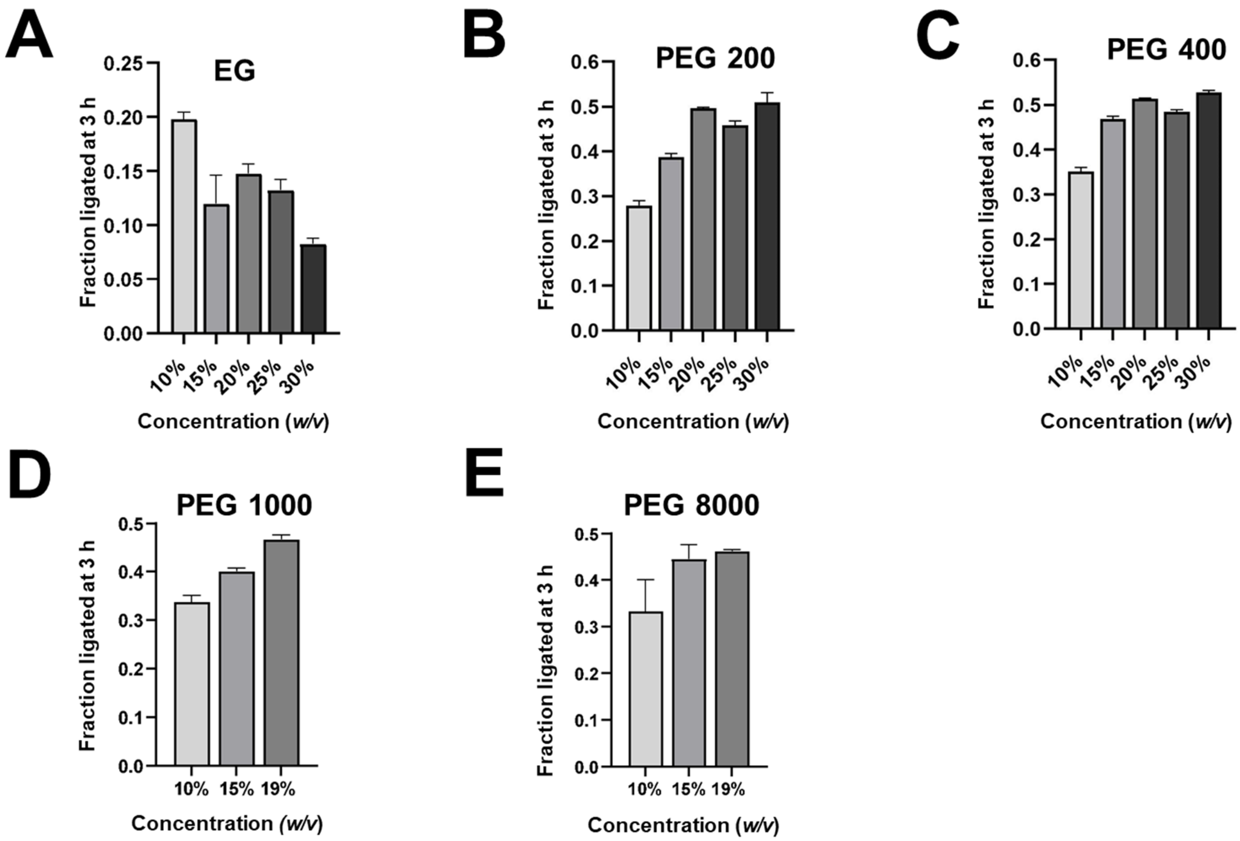

Figure S1. Identification of optimal crowding conditions for ligase 1-catalyzed RNA ligation. Fraction ligated after 3 h in the presence of (A) 10-30% ethylene glycol (EG), (B) 10-30% polyethylene glycol (PEG) 200, (C) 10-30% PEG 400, (D) 10-19% PEG 1000, (E) 10-19% PEG 8000. Ligation reactions contained 1 μM ribozyme, 1.2 μM RNA template, and 2 μM 2-AI-activated RNA substrate in 100 mM Tris-HCl pH 8.0 and 1 mM $MgCl_2$. Reactions contained additives (EG, PEG 200-8000) as indicated.

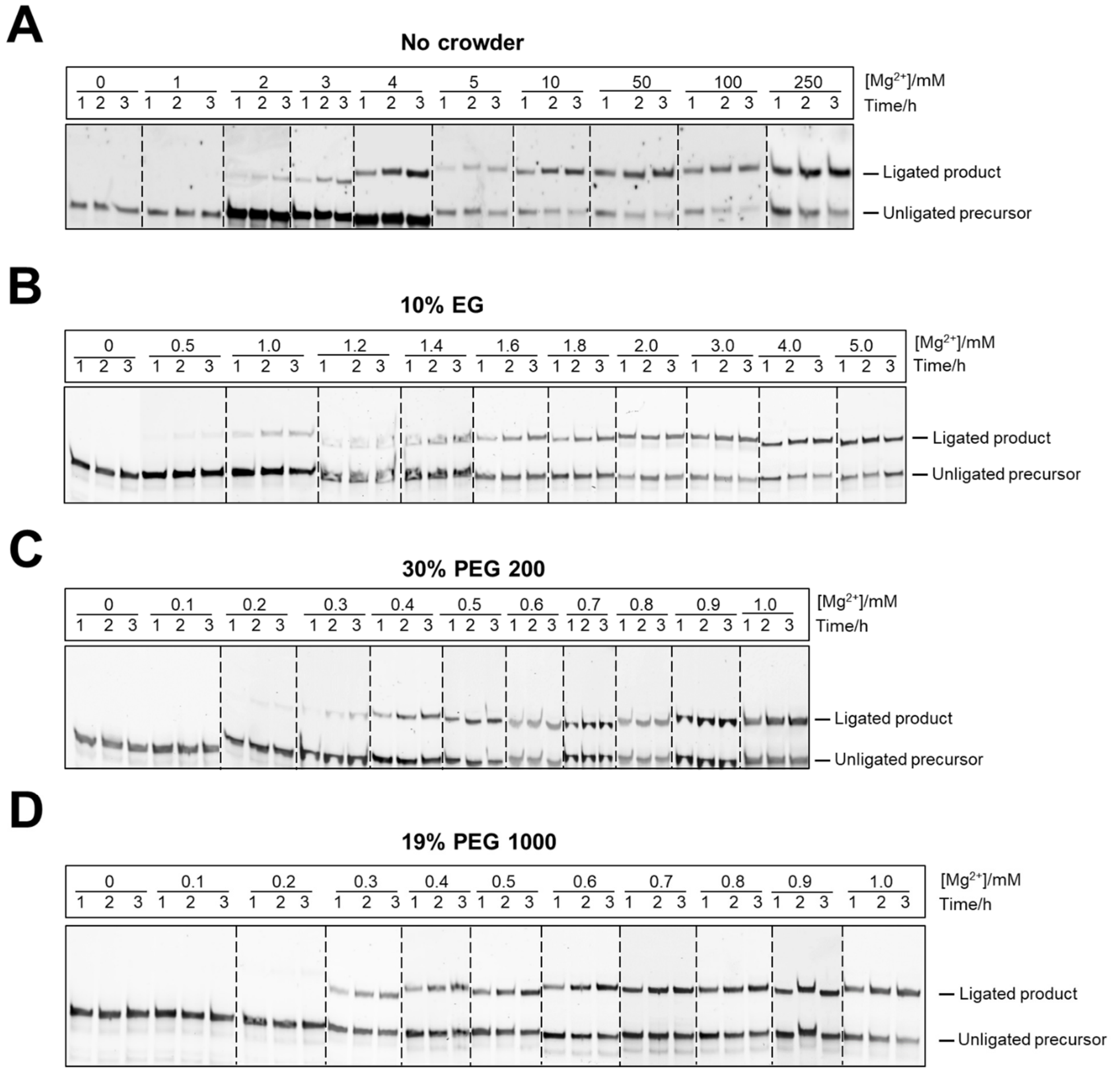

Figure S2. Representative gels illustrating the Mg^{2+} dependence of ligase 1 ribozyme-catalyzed RNA ligation in the absence and presence of crowding agents. (A) No crowding agents. (B) 10% EG. (C) 30% PEG 200 (D) 19% PEG 1000. Ligation reactions contained 1 μM ribozyme, 1.2 μM RNA template, and 2 μM 2-AI-activated RNA substrate in 100 mM Tris-HCl pH 8.0 and the indicated concentrations of $MgCl_2$. Reactions contained additives as indicated.

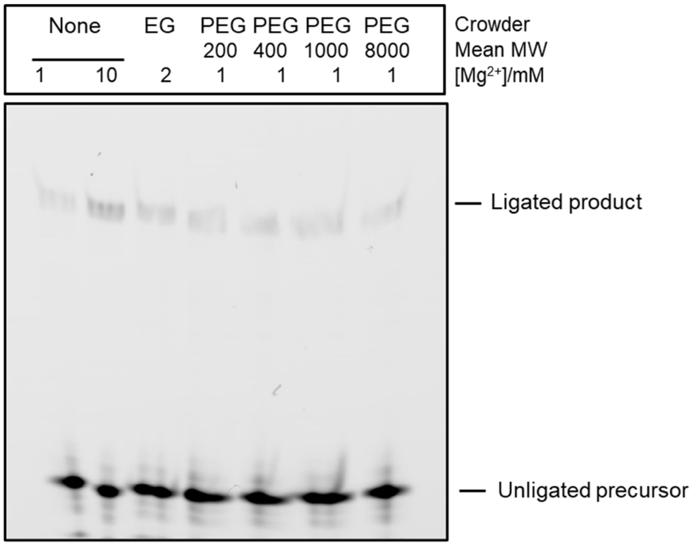

Figure S3. Nonenzymatic ligation is not influenced by molecular crowding. EG or PEGs do not rescue template-directed nonenzymatic RNA ligation at low Mg^{2+} concentrations. Ligation reactions contained 1 μM FAM-labeled RNA primer, 1.2 μM RNA template, and 2 μM 2-AI-activated RNA substrate in 100 mM Tris-HCl pH 8.0 and 1 mM, 2 mM, or 10 mM $MgCl_2$. Reactions contained additives (10% EG, 30% PEG 200, 30% PEG 400, 19% PEG 1000, or 19% PEG 8000) as indicated. None indicates the absence of crowders.

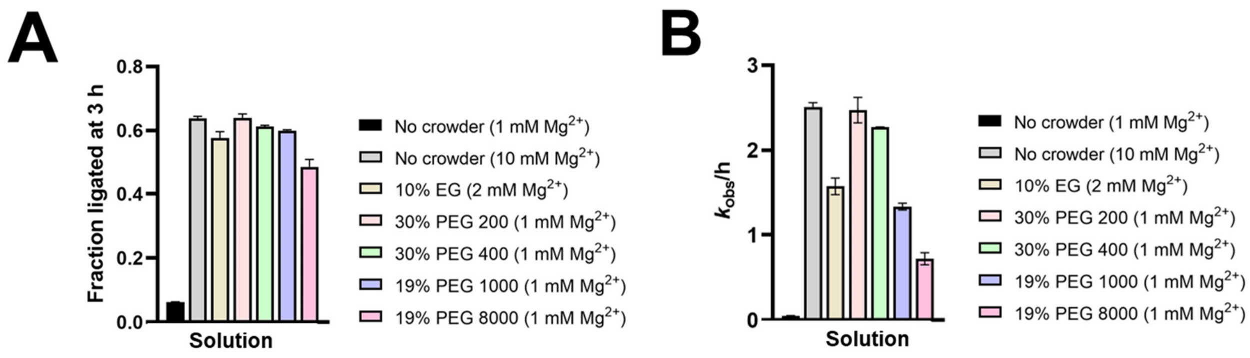

Figure S4. Ligase 2 activity at low Mg^{2+} concentrations is rescued by crowding agents. In the presence of 1-2 mM Mg^{2+}, crowding agents stimulate (A) ligation yields and (B) ligation rates. Ligation reactions contained 1 μM ribozyme, 1.2 μM RNA template, and 2 μM 2-AI-activated RNA substrate in 100 mM Tris-HCl pH 8.0 and the indicated concentrations of $MgCl_2$ (1 mM, 2 mM, or 10 mM). Reactions contained additives (EG, PEG 200-8000) as indicated.

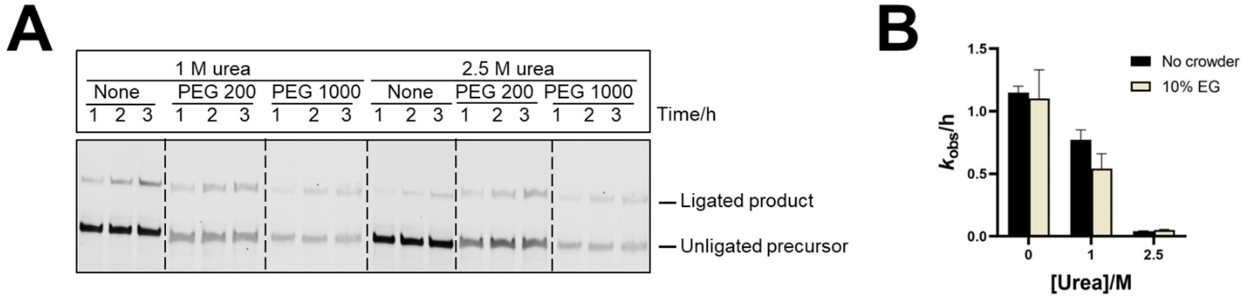

Figure S5. Effect of molecular crowding on ligase 3-catalyzed RNA ligation in the presence of urea. (A) Ligase 3 activity in the presence of urea, in the absence of crowding, or in the presence of either 30% PEG 200 or 19% PEG 1000. (B) 10% EG does not impart any beneficial effect to ligation in the presence of urea. Ligation reactions contained 1 μM ribozyme, 1.2 μM RNA template, and 2 μM 2-AI-activated RNA substrate in 100 mM Tris-HCl, pH 8.0, 1 mM (A) or 2 mM (B) $MgCl_2$ in the presence or absence of urea. Reactions contained additives as indicated. None indicates the absence of crowding agents.

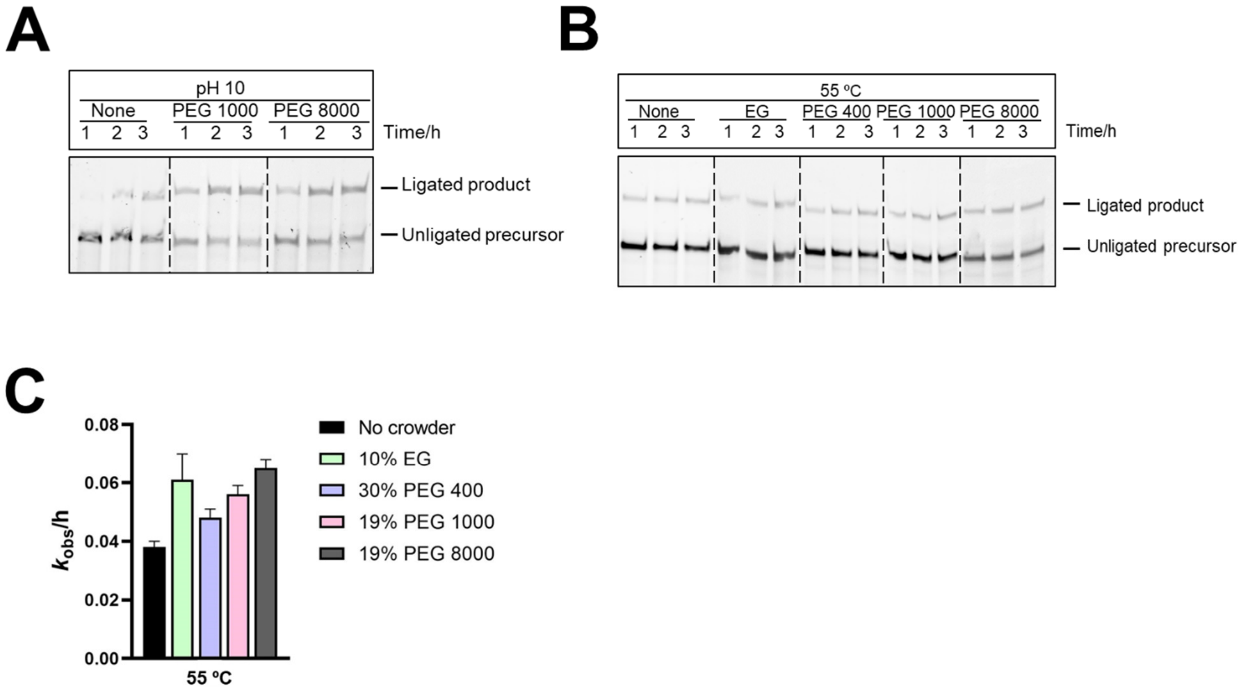

Figure S6. Effect of molecular crowding on ligase 3-catalyzed RNA ligation under alkaline pH and high temperature. (A) Ligase 3 activity at pH 10 in the absence or presence of crowding agents. (B) Ligase 3 activity at 55 °C in the absence or presence of crowding agents. (C) Ligase 3 exhibits a modest increase in ligation rates upon the addition of EG and PEG at 55 °C in the presence of 1 mM Mg^{2+}. Ligation reactions contained 1 μM ribozyme, 1.2 μM RNA template, and 2 μM 2-AI-activated RNA substrate in 100 mM buffer: CAPS pH 10 (A) or Tris-HCl pH 8.0 (B). Reactions contained additives (10% EG, 30% PEG 400, 19% PEG 1000, or 19% PEG 8000) as indicated. None indicates the absence of crowding agents.

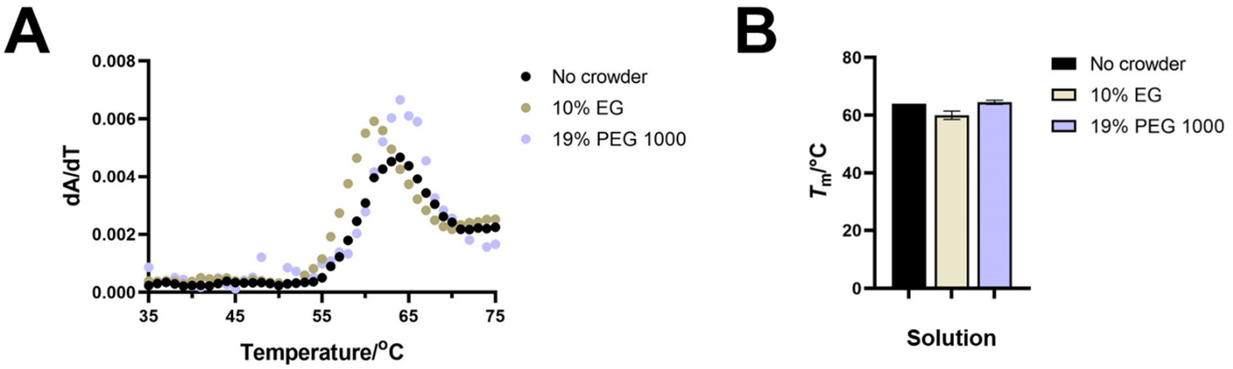

Figure S7. Effect of molecular crowding on the thermal stability of the ligase 1 ribozyme. (A) Thermal denaturation of ligase 1 measured by UV spectroscopy in 1 mM Mg^{2+}. Absorbance was recorded at 260 nm. $(dA/dT)_{max}$ occurs around the same temperature in both the uncrowded sample and in the sample with PEG 1000. $(dA/dT)_{max}$ was left-shifted in the presence of EG. (B) The melting temperature (T_m) of ligase 1 in an uncrowded solution was comparable to the T_m values observed in the presence PEG 1000, but T_m fell by ~4 °C in the presence of EG. Thermal denaturation was performed with 0.5 μM ribozyme in 10 mM sodium cacodylate buffer, pH 7.

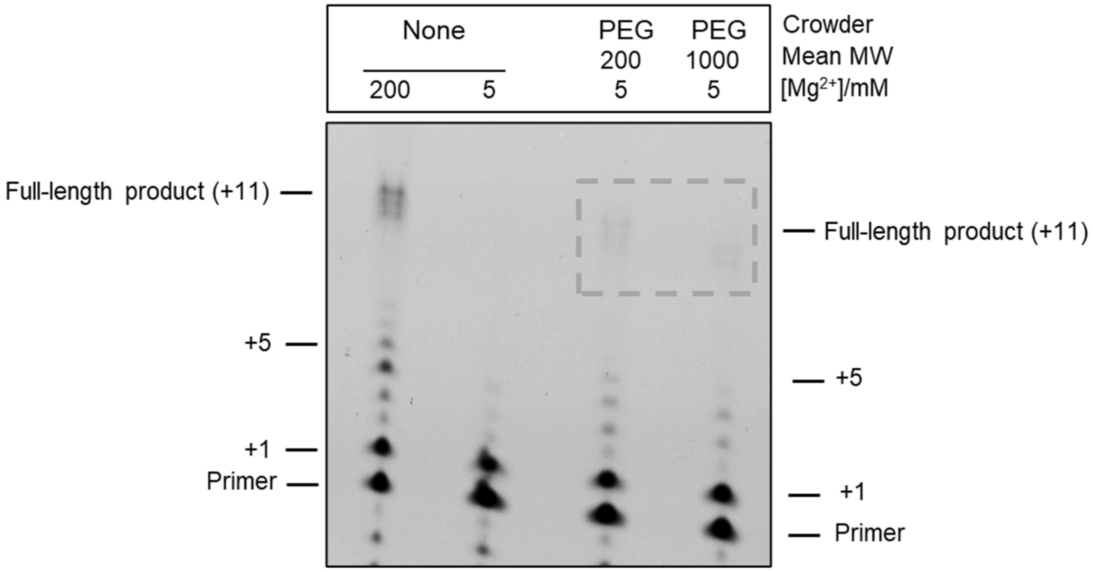

Figure S8. RNA-catalyzed polymerization of NTPs at low Mg^{2+} concentration. Ribozyme (38-6 polymerase)-catalyzed full-length polymerization product was detected at 5 mM Mg^{2+} in reactions containing 30% PEG 200 and 19% PEG 1000; however, no such product was detected after 24 h in an uncrowded solution containing 5 mM Mg^{2+}. At 5 mM Mg^{2+}, crowders increased the fraction of extended primer from 26% in uncrowded solution to ~40%. Polymerization reactions containing a FAM-labeled RNA primer (80 nM), RNA template (100 nM), 2 mM total NTPs, and polymerase ribozyme (100 nM) in 25 mM Tris-HCl, pH 8 and 5 mM or 200 mM Mg^{2+} were incubated at 17 °C. None indicates the absence of crowders.

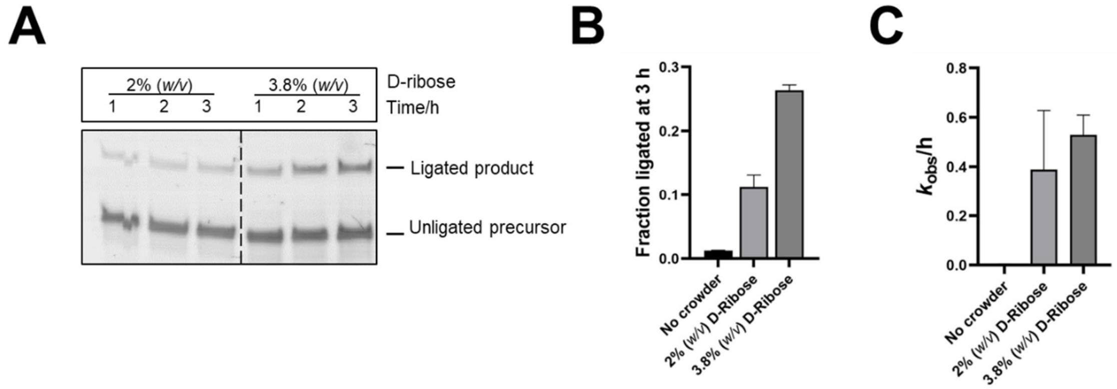

Figure S9. Ribozyme-catalyzed RNA ligation is stimulated in the presence of ribose. (A) Ligase 1 ribozyme is active at 1 mM Mg^{2+} in the presence of 2% and 3.8% (*w/v*) D-ribose. (B) Ligation yields after 3 h at 1 mM Mg^{2+} increased to 11% and 26% from ~1% in the presence of 2% and 3.8% ribose, respectively. (C) Ligation rates were enhanced significantly in the presence of 2% and 3.8% ribose. Ligation reactions contained 1 μM ribozyme, 1.2 μM RNA template, and 2 μM 2-AI-activated RNA substrate in 100 mM Tris-HCl pH 8.0 and 1 mM $MgCl_2$. Reactions were performed in the absence or presence of D-ribose.

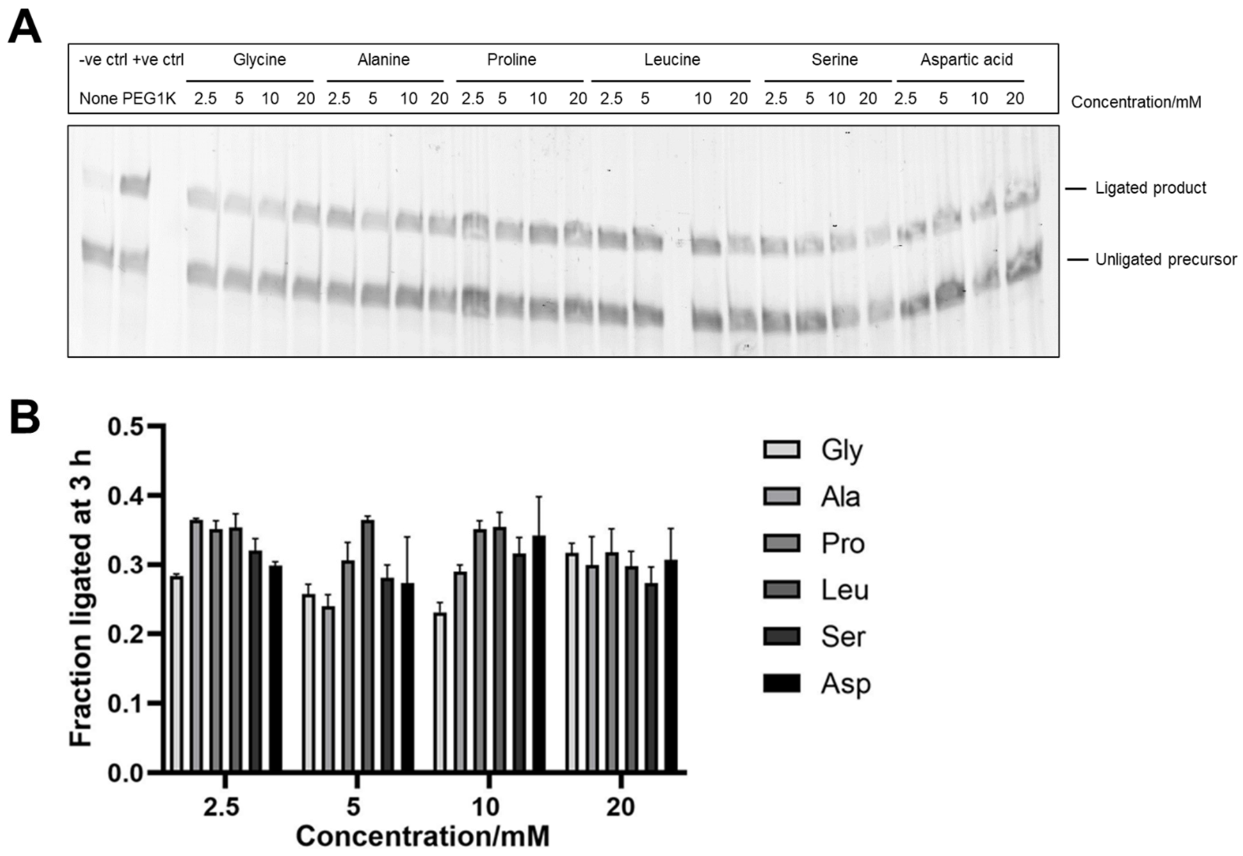

Figure S10. Ribozyme-catalyzed RNA ligation is stimulated in the presence of prebiotically relevant amino acids. (A) Ligase 1 ribozyme is active at 1 mM Mg^{2+} in the presence of 2.5 - 20 mM amino acids (Gly, Ala, Pro, Leu, Ser, Asp). 19% PEG 1000 was used as a positive control. (B) Ligation yields after 3 h were comparable across the different concentrations tested. Ligation reactions contained 1 μM ribozyme, 1.2 μM RNA template, and 2 μM 2-AI-activated RNA substrate in 100 mM Tris-HCl pH 8.0 and 1 mM $MgCl_2$. Reactions were performed in the absence or presence of the indicated amino acids. None indicates the absence of crowders.
