# Supplementary material for: Molecular Crowding Facilitates Ribozyme-Catalyzed RNA Assembly
Source: ACS Cent Sci. 2023 Aug 3;9(8):1670–8. doi: 10.1021/acscentsci.3c00547 (PMC10451029; doi:10.1021/acscentsci.3c00547)
Supplement: Supplementary file 2 — oc3c00547_si_002.pdf [file oc3c00547_si_002.pdf]

oc-2023-00547w.R1

Name: Peer Review Information for "Molecular crowding facilitates ribozyme-catalyzed RNA assembly"

First Round of Reviewer Comments

Reviewer: 1

Comments to the Author

This is a really interesting manuscript that suggests a plausible solution to the 'magnesium ion problem'. This problem relates to the requirement for high magnesium ion concentrations for ribozyme folding and function, but low concentrations for maintenance of fatty acid vesicles. The solution suggested is crowding and this explored by a thorough experimental investigation using both prebiotically implausible polyethyleneglycols and prebiotically plausible sugars and amino acids. In short, crowding reduces the need for high concentrations of magnesium ions. The effects of crowding are not huge, but they look to be close to enough to get round the magnesium ion problem and, as such, this manuscript certainly deserves to be published in a high quality journal such as ACS Central Science. I couldn't see any typos or other errors and my only suggested change is to bring the explanation as to what [Mg2+] 1/2 is to the first mention of it.

Reviewer: 2

Comments to the Author

It has been suggested that ribozyme was a key material of the origin of life. However, ribozymes usually need a high concentration of Mg2+ for the activities compared to physiological Mg2+ concentration. To address this gap, the authors investigated the effect of molecular crowding on the activity of RNA ligase ribozymes under low Mg2+ conditions and found that molecular crowding drastically improved the activity of those ribozymes at low Mg2+ conditions. Thus, they concluded that molecular crowding had a key role in the evolution of the origin of life. The authors conducted ribozyme assays under various crowding conditions with different ribozymes for RNA assembly to demonstrate a general mechanism of the activation of the ribozyme reactions. However, there are three fundamental issues for publication in this journal.

1) Many studies have already shown that molecular crowding accelerated the activity of ribozymes at low Mg2+ concentrations. For example, the study cited as ref 26 in this manuscript was similar. The study of ref 26 demonstrated the ribozyme assays under low Mg2+ conditions with various molecular crowdings in the absence or presence of urea and concluded that molecular crowding facilitated the reactions, although the study focused on ribozymes for RNA hydrolysis. The same group also found the activation of ribozyme by amino acids under low Mg2+ conditions (Nat Commun 9, 2149 (2018)). In the case of RNA assembly reaction, another group already found that molecular crowding accelerated the

activity of RNA polymerase ribozyme at 2 mM Mg2+ condition (Biochemistry, 58, 8, 1081 (2019)). Therefore, the work in this study does not have a novel impact.

2) The mechanism of ribozyme of activation by molecular crowding is not clear. The authors should demonstrate how molecular crowding facilitated the reaction of RNA assembly by a unique mechanism different from other ribozymes reported before. The authors indirectly claimed that the effect of crowding on the ribozyme structure was a key without direct evidence, but this is the same scenario as previous findings. More physical studies are needed such as structural analyses and quantitative treatments according to the physicochemical properties of solution.

3) Although this manuscript seems to be a systematic study of the effect of crowders on ribozyme activity, the authors' story was based on a one-side view. For example, in Figure 1, the authors compared results obtained in 10%EG with 2 mM Mg2+, 30% PEG200 with 1 mM Mg2+, 19% PEG8000 with 1 mM Mg2+, and so on. How can readers compare the data that was obtained in different concentrations and conditions? Other similar points are listed later.

Therefore, this reviewer thinks that this manuscript is preliminary to be publishable in this journal.

<Other comments>

1. The description of Fig. 1D is missing, and Fig S8 was misguided as S7 in the text.

2. As shown in Figure S1, EG showed a trend of decrease in activity with increasing EG concentration. What is the difference between EG and other crowders? As commented above, the effect on the activation depending on the crowder size is unclear.

3. In the assays under denaturation conditions, why did the authors use 30% PEG200 and 19 % PEG1000 for urea and 19% PEG1000 and 19% PEG8000 for high pH? This is unsystematic and unfair.

4. In the UV melting assay, the authors concluded there was a negligible increase in thermal stability in both EG and PEG1000. However, the result indicates that 10% EG obviously decreased the stability, whereas 19% PEG1000 did increase. These conflicting results should not be ignored and must indicate the different mechanisms for the activation of the ribozyme reaction depending on the crowder size.

5. In the paragraph of prebiotically relevant small molecules, the authors should discuss the difference of the mechanism between crowders and amino acids quantitatively.

Reviewer: 3

Comments to the Author

oc-2023-00547w

In this manuscript, DasGupta et al conduct a survey to test the activity rescue ability of molecular crowding on ribozymes. They used four different ribozymes, including three ligases and one polymerase to demonstrate that the molecular crowding environment formed by PEG can help some ligase or polymerase ribozymes to regain activity under low salt, high pH, high temperature, or high urea. The authors also demonstrated that prebiotically small molecules such as ribose and amino acids can also provide a similar beneficial effect as PEG.

It has been known for more than one decade that molecular crowding stabilizes ribozyme structure, changes folding pathways, and enhances activity. The reported data here expanded the stabilization effect of crowding from less Mg^{2+} dependence to the tolerance of denaturing environment. The authors also include other more life-relevant crowding agents such as amino acids and ribose. Although it is interesting to see that crowing agents can help ribozymes to overcome various negative impact factors, and various crowding agents can achieve similar stabilization effects on salt dependence, there are no alternative methods other than activity assay in this manuscript to support the finding. This weakness leads to insufficient data to support any mechanism of stabilization proposed in the article. The rationality behind ribozyme selection was not well plotted. The ambiguity of some descriptions and misciting the data figure also weaken the work.

The main criticism from this reviewer is as follows:

To show the beneficial effect of crowding as a general phenomenon, more ribozymes with a larger diversity of sequence should be screened. Or at least all the ribozymes used in the study should be tested with each of the negative affecters that were proposed to be overcome by crowding agents. For example, it is questionable why the authors choose not to use ligase 1&2 in the denaturation section. It was stated that s 1 and 2 were inactive at low Mg^{2+} in the absence of crowding agents. But why did the authors not perform the assay at an intermediate Mg^{2+} concentration that can show both crowding compensation for Mg^{2+} and denaturation? It is also unclear if ligase 3 Mg^{2+} dependence can be rescued by PEG as ligase 1&2.

The minor suggestions from this reviewer are as follows:

1. Fig 1D was not mentioned in the main text.
2. PEG 400 has the highest Kobs in Fig.1 but was not discussed specifically.
3. Page 6, lines 10-13, the authors proposed an alternative explanation to the induction of RNA folding, but no folding pattern of the ribozyme has been described in this article. They only mentioned activity.
4. Fig. S3 has no crowder concentration. It is also hard to say if the crowder has no effect because the ligation efficiency of the nonenzymatic ligation is low to start from.
5. Why does S5 use two different [MgCl2] (1 mM in S5A and 2 mM in S5B)?
6. The term “nucleic acid assembly”, especially “assembly”, was used in an unclear manner. For example, on Page 8, line 51, the authors wrote “ribozyme-catalyzed RNA assembly” on RNA polymerase activity.
7. Page 8, line 48 should be Fig S8

Reviewer: 4

Comments to the Author

The manuscript by DasGupta et al. reports molecular crowding-induced stimulation of RNA-catalyzed RNA ligation and polymerization. The authors studied the catalytic activities of several ligase and

polymerase ribozymes at low Mg ion concentrations. RNA ligation assays showed enhanced ligation rates and decreased Mg ion requirements for RNA folding in the presence of crowders, EG and PEGs of various MWs. The presence of the crowders protected the ribozyme from unfolding in the presence of high concentrations of urea and in alkaline pH solutions. Prebiotically relevant small molecules such as EG, ribose, and amino acids also facilitated the ligation. The authors propose an important role of molecular crowding in the evolution of RNA-based primordial life. Overall, investigation of the ribozyme activity in crowed environments is very important. This study is carefully designed and the conclusion is reasonable. However, the results of enhanced ribozyme activities in crowed environments are not very surprising (crowding-induced RNA folding and catalysis have already been reported for several types of ribozymes although this study uses ligase and polymerase ribozymes that are relevant to the evolution of earliest living cells). This study appears too specialized to be of interest to the readers of ACS Central Science, and it would be better fit to a more specialized journal.

I have several other concerns as follows: (1) The mechanisms that stimulate the ribozyme activities in the presence of both large and small molecules are not deeply discussed. In particular, how can amino acids stimulate the ligation? (2) The experiments were conducted at low salt concentrations. However, the amounts of monovalent metal ions such as K and Na ions may not be low in prebiotic environments. RNA base-pairing and folding are strongly influenced by monovalent metal ions when divalent metal ions are absent or very low. Further studies using buffer solutions containing moderate concentrations of monovalent metal ions are needed to draw definite conclusions. Does the authors use salt-fee amino acid solutions? Unless, counter cations of amino acids may affect the efficiency of RNA folding at low Mg ion concentrations. I would like to see the results in the presence of NaCl in place of amino acids, in Figure 4. (3) Based on the ligation yields, I suppose the reaction does not complete (the fraction of active complex is not close to 1.0). Are the kinetic data for RNA-catalyzed reactions fit to a single exponential function without a burst phase? (4) UV melting experiments were conducted with the ligase ribozyme. However, the complex formation between the ribozyme, substrate, and template is crucial for the preservation of ribozyme activity. It would be best to measure the thermal stability of the RNA complex. (5) It is better to mention the quantitativity of RNA stain using SYBR Gold. The extent of fluorescence enhancement upon SYBR Gold binding to RNA is not the same between the unligated precursor and ligated product because of different RNA lengths. (6) On page 5, line 11: the amount of PEG400 is missing, which is probably 30%. (7) The reaction temperature for ligation assays is not stated. Concentrations of ribose and amino acids for the data of Figure 4 are not described.

Author's Response to Peer Review Comments:

Attached: Cover letter with responses to reviewers' comments

July 6, 2023

Professor Editor
Editor,
ACS Central Science

Dear Professor Editor,
This letter accompanies the submission of the revised version of our manuscript "*Molecular crowding facilitates ribozyme-catalyzed RNA assembly*" by Saurja DasGupta, Stephanie J. Zhang, and Jack W. Szostak. We have modified the manuscript to carefully address all the reviewers' comments and included point-by-point responses for all comments, as detailed below. We hope that you will find our revised manuscript suitable for publication in *ACS Central Science*.

Sincerely,

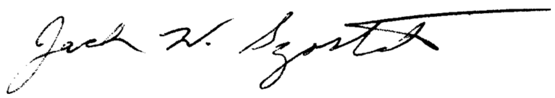

Jack W. Szostak
Investigator, Howard Hughes Medical Institute
Professor of Chemistry
The University of Chicago

RESPONSE TO REVIEWERS' COMMENTS

We thank all four reviewers for their time and valuable suggestions that, we hope, have considerably improved our manuscript.
Responses to each comment are given below.

Reviewer: 1

Comments:
This is a really interesting manuscript that suggests a plausible solution to the 'magnesium ion problem'. This problem relates to the requirement for high magnesium ion concentrations for ribozyme folding and function, but low concentrations for maintenance of fatty acid vesicles. The solution suggested is crowding and this explored by a thorough experimental investigation using both prebiotically implausible polyethylene glycols and prebiotically plausible sugars and amino acids. In short, crowding reduces the need for high concentrations of magnesium ions. The effects of crowding are not huge, but they look to be close to enough to get round the magnesium ion problem and, as such, this manuscript certainly deserves to be published in a high quality journal such as ACS Central Science. I couldn't see any typos or other errors and my only suggested change is to bring the explanation as to what [Mg2+] 1/2 is to the first mention of it.

We thank the reviewer for their enthusiasm for this work and for appreciating the significance of this research.
We have now described what $[Mg^{2+}]_{1/2}$ means in the second paragraph of the 'Introduction' where we first mention it in the manuscript:

"For example, the Mg^{2+} concentration at which half-maximum ligation rate was achieved, $[Mg^{2+}]_{1/2}$, of the first-of-its-kind, class I ligase is 70-100 mM[8] and polymerase ribozymes derived from the class I ligase have an optimal $[Mg^{2+}]$ of ~200 mM.[9, 10]"

Reviewer: 2

Comments:

It has been suggested that ribozyme was a key material of the origin of life. However, ribozymes usually need a high concentration of Mg2+ for the activities compared to physiological Mg2+ concentration. To address this gap, the authors investigated the effect of molecular crowding on the activity of RNA ligase ribozymes under low Mg2+ conditions and found that molecular crowding drastically improved the activity of those ribozymes at low Mg2+ conditions. Thus, they concluded that molecular crowding had a key role in the evolution of the origin of life. The authors conducted ribozyme assays under various crowding conditions with different ribozymes for RNA assembly to demonstrate a general mechanism of the activation of the ribozyme reactions. However, there are three fundamental issues for publication in this journal.

1) Many studies have already shown that molecular crowding accelerated the activity of ribozymes at low Mg2+ concentrations. For example, the study cited as ref 26 in this manuscript was similar. The study of ref 26 demonstrated the ribozyme assays under low Mg2+ conditions with various molecular crowdings in the absence or presence of urea and concluded that molecular crowding facilitated the reactions, although the study focused on ribozymes for RNA hydrolysis. The same group also found the activation of ribozyme by amino acids under low Mg2+ conditions (Nat Commun 9, 2149 (2018)). In the case of RNA assembly reaction, another group already found that molecular crowding accelerated the activity of RNA polymerase ribozyme at 2 mM Mg2+ condition (Biochemistry, 58, 8, 1081 (2019)). Therefore, the work in this study does not have a novel impact.

We agree that the observation that molecular crowding stimulates ribozyme activity is not new. However, the novel and significant aspect of our work is to show that molecular crowding in general, and in particular using prebiotically plausible reagents, can lower the Mg^{2+} requirement of ribozymes that synthesize or assemble RNA and are thus relevant to the origin and evolution of early life. To our knowledge, only two reviews previously touch on this point: Saha et al., 2014, Orig. Life Evol. Biosph.; DasGupta et al., 2020, Org. Biomol. Chem. The incompatibility of Mg^{2+} with the hydrolytic stability of activated RNA building blocks and fatty acid-based primitive cell membranes poses a significant roadblock toward the assembly of complex, functional RNAs and the functioning of ribozymes within model fatty acid protocells. Therefore, addressing this issue is important. As the reviewer mentions, a large majority of previous studies involving crowding and ribozyme activity have been done on self-cleaving ribozymes that already do not have high Mg^{2+} requirements. We thank the reviewer for pointing us to the Takahashi et al., 2019, Biochemistry paper that reports PEG 200-assisted rate stimulation of the tC9Y polymerase ribozyme, which we now include as a reference. We used a different ribozyme, the 38-6 polymerase, in our experiments. Our results with the 38-6 polymerase ribozyme are consistent with the results obtained with the tC9Y ribozyme, further

strengthening the generality of molecular crowding as mechanism to lower the Mg^{2+} requirements of ribozymes.

2) The mechanism of ribozyme of activation by molecular crowding is not clear. The authors should demonstrate how molecular crowding facilitated the reaction of RNA assembly by a unique mechanism different from other ribozymes reported before. The authors indirectly claimed that the effect of crowding on the ribozyme structure was a key without direct evidence, but this is the same scenario as previous findings. More physical studies are needed such as structural analyses and quantitative treatments according to the physicochemical properties of solution.

We do not claim that the crowding-mediated activity enhancement of ligase and polymerase ribozymes discussed in this work follows a mechanism that is distinct from other instances of crowding-induced ribozyme activation, such as for self-cleaving ribozymes. Ribozyme rate stimulation is most likely caused due to an increase in the fraction of folded RNA molecules in the presence of crowders. The lack of a beneficial effect of crowding on nonenzymatic ligation suggests the importance of folded RNA structures. The rescue of ligation in the presence of a denaturant, urea, also indirectly suggests that crowders promote RNA folding. Enhanced ribozyme folding reduces the solvent-accessible surface area of the RNA which minimizes interactions with urea and preserves activity. As briefly discussed in the Introduction, folding may be promoted by entropic forces of volume exclusion in the presence of high MW crowders like PEG 1000 or PEG 8000, or enthalpic forces such as direct interactions between the crowder and the RNA surface, and altered solvent properties like dielectric constant, osmotic pressure, or viscosity. While low MW crowders do not exclude significant volume and hence have minimal entropic contributions to crowding, both low MW and high MW crowders show enthalpic effects. Therefore, in most cases, the observed rate stimulation is a result of a complex interplay between various forces that is difficult to disentangle without a great deal of additional experimentation and computational simulations.

The primary goal of our work is to introduce molecular crowding as a potential solution to the Mg^{2+} problem of ribozyme-catalyzed RNA assembly, and more generally the functioning of ribozymes relevant to the origin and evolution of early life. More involved experimentation directed at investigating the details of how crowders modify the structure and function of ribozymes is beyond the scope of this study.

3) Although this manuscript seems to be a systematic study of the effect of crowders on ribozyme activity, the authors' story was based on a one-side view. For example, in Figure 1, the authors compared results obtained in 10%EG with 2 mM Mg2+, 30% PEG200 with 1 mM Mg2+, 19% PEG8000 with 1 mM Mg2+, and so on. How can readers compare the data that was obtained in different concentrations and conditions? Other similar points are listed later.

We thank the reviewer for the opportunity to clarify this point. Fig. 1 B-D shows ribozyme activity at 1 mM Mg^{2+} for a series of PEGs and 2 mM Mg^{2+} for EG. This was done to capture the optimal effect of these crowders. We found from Mg^{2+} titration experiments (Fig. 2) that ligation rates plateaued at 2 mM Mg^{2+} for EG, but just under 1 mM Mg^{2+} for PEGs. The yield and rate constant data for 2 mM Mg^{2+} in the absence of crowding is presented in Fig. 1 C, D to make the comparison between 'no crowder' and '+EG' ligations more accurate and meaningful.

Although ligation at 1 mM Mg^{2+} is suboptimal in the presence of EG, in Fig. S1A, we show 20% ligation yield at 1 mM Mg^{2+} with EG, but negligible ligation in the absence of crowders. We have added the following sentence at the end of the second paragraph of the 'Results and Discussion' section to clarify this:

"Because EG shows optimal activity at 2 mM Mg^{2+}, all experiments with EG (except for the screening experiment in Fig. S1 and the ligation experiment at 55 ºC) were performed at 2 mM Mg^{2+}. "

Therefore, this reviewer thinks that this manuscript is preliminary to be publishable in this journal.

<Other comments>
1. The description of Fig. 1D is missing, and Fig S8 was misguided as S7 in the text.

We thank the reviewer for noticing this. We have now corrected these errors.

2. As shown in Figure S1, EG showed a trend of decrease in activity with increasing EG concentration. What is the difference between EG and other crowders? As commented above, the effect on the activation depending on the crowder size is unclear.

We agree that the mechanism by which each crowder activates RNA catalysis at low [Mg^{2+}] is difficult to ascertain. Due to its small size, EG likely exerts minimal volume exclusion, and its crowding effects are probably due to enthalpic interactions directly with the RNA or through altered solvent properties. Increasing EG concentration above the optimal level may hinder productive collisions between molecules through increased solution viscosity, thereby attenuating ligation. We added the following sentences at the end of the first paragraph of the 'Results and Discussion' section to address this issue:

"Ligation yield decreased with an increase in the concentration of EG above 10%. This trend is different from other PEG-based crowders which exhibit better ligation at higher concentrations (Fig. S1). This difference between EG and PEGs could be due to the mechanism in which these crowders effect RNA structure. EG cannot exclude significant volume due to its small size and must act through direct interactions with the RNA backbone or through solvent effects which increase the association

between RNA and Mg^{2+}. Therefore, the EG crowding effects observed are likely enthalpic in contrast to the entropic contributions from PEGs, especially ones with moderate to high MWs.

3. In the assays under denaturation conditions, why did the authors use 30% PEG200 and 19 % PEG1000 for urea and 19% PEG1000 and 19% PEG8000 for high pH? This is unsystematic and unfair.

We used PEG 200 and PEG 1000 to represent low MW and high MW crowders, respectively for denaturation experiments with urea. When we tested the effect of PEG 200 on RNA ligation at pH 10, we did not observe significant rate enhancement for PEG 200 relative to the 'no crowder' control. Another low MW species, EG, similarly provided no benefit. But the high MW crowder, PEG 1000 exhibited a beneficial, albeit modest, effect at pH 10, which was recapitulated by another high MW crowder, PEG 8000. For this reason, we used PEG 200 and PEG 1000 for denaturation experiments and PEG 1000 and PEG 8000 for experiments at pH 10.

We have now added the following sentence (Subsection: 'Crowding protects ligase ribozyme from denaturation', paragraph 2, line 8) to reflect these observations:

"We tested ligation at pH 10 with different crowders. Low MW crowders like EG and PEG 200 showed no benefit; however, the loss of ligase activity at pH 10 was less pronounced in the presence of high MW crowders, PEG 1000 and PEG 8000 with only a 2.6-fold and 2.3-fold reduction in k_{obs}, respectively relative to their values at pH 8 (Fig. 3B, Fig. S6A)."

4. In the UV melting assay, the authors concluded there was a negligible increase in thermal stability in both EG and PEG1000. However, the result indicates that 10% EG obviously decreased the stability, whereas 19% PEG1000 did increase. These conflicting results should not be ignored and must indicate the different mechanisms for the activation of the ribozyme reaction depending on the crowder size.

We now discuss the difference in the effects of EG and PEG 1000 on the thermal stability of the ligase ribozyme. As the reviewer rightly pointed out, EG caused a 4 ºC decrease in T_m, while the presence of PEG 1000 resulted in an apparent 0.5 ºC increase, which is not statistically significant. A decrease in thermal stability caused by EG has been previously observed with the hammerhead ribozyme (Nakano et al. 2015, ChemBioChem). Interaction with EG might destabilize base-paired helices resulting in a fall in T_m. We have modified the relevant text as follows:

"The lack of substantial benefit from crowding at high temperatures is consistent with UV melting experiments with ligase 1 ribozyme, which revealed a negligible increase (ΔT_m = 0.5 ºC) in its thermal

stability in the presence of the high MW crowder, PEG 1000 (Fig. S7A, B). EG, on the other hand, caused a 4 ºC decrease in T_m (Fig. S7A, B). A similar decrease in the T_m value in the presence of EG has been observed with the hammerhead ribozyme, which we speculate could be due to a destabilization of base-paired helices.[31]"

5. In the paragraph of prebiotically relevant small molecules, the authors should discuss the difference of the mechanism between crowders and amino acids quantitatively.

The mechanism by which ribozyme-catalyzed ligation is enhanced by amino acids is not entirely clear. It was suggested by Yamagami et al. (Nat. Commun. 2018) that amino acids, similar to crowders like PEGs, induce structural compaction in RNAs. Thermal denaturation and SAXS studies on self-cleaving ribozymes supported this claim. In the absence of volume exclusion, amino acids may alter solvent properties. Additionally, by weakly chelating Mg^{2+}, amino acids may form a layer on the RNA surface where Mg^{2+} is shared between the amino acids and the RNA backbone, thereby increasing the local concentration of Mg^{2+}. We now note these possibilities in the following paragraph with appropriate citations:

"The mechanism of ribozyme activation at low Mg^{2+} by ribose or amino acids is not clear. Aliphatic alcohols such as methanol, ethanol, propanol, 2-methoxyethanol, and propane-1,3-diol stimulate hammerhead catalysis at 1 mM Mg^{2+} by decreasing the dielectric constant of the solution, thereby enhancing interactions between the ribozyme and Mg^{2+}.[31] Ribose-mediated enhancement of ribozyme-catalyzed RNA ligation could be a result of similar solution-level effects. The beneficial effect of amino acids on ribozyme activity has been previously observed for RNA self-cleavage. It was proposed that the increase in ribozyme activity resulted from structural compaction of the RNA which allowed greater sampling of its catalytic fold.[40] This assertion was supported by thermal denaturation and SAXS studies. Amino acids may stimulate RNA folding by altering solvent properties like dielectric constant or water activity.[17] Additionally, as amino acids can weakly chelate Mg^{2+}, the chelated amino acids may form a layer on the RNA surface, increasing the local concentration of Mg^{2+}, which may lead to improved folding and catalysis.[40, 41]"

Reviewer: 3

Comments:

In this manuscript, DasGupta et al conduct a survey to test the activity rescue ability of molecular crowding on ribozymes. They used four different ribozymes, including three ligases and one polymerase to demonstrate that the molecular crowding environment formed by PEG can help some ligase or polymerase ribozymes to regain activity under low salt, high pH, high temperature, or high urea. The authors also demonstrated that prebiotically small molecules such as ribose and amino acids can also provide a similar beneficial effect as PEG. It has been known for more than one decade that molecular crowding stabilizes ribozyme structure, changes folding pathways, and enhances activity. The reported data here expanded the stabilization effect of crowding from less Mg2+ dependence to the tolerance of denaturing environment. The authors also include other more life-relevant crowding agents such as amino acids and ribose. Although it is interesting to see that crowing agents can help ribozymes to overcome various negative impact factors, and various crowding agents can achieve similar stabilization effects on salt dependence, there are no alternative methods other than activity assay in this manuscript to support the finding. This weakness leads to insufficient data to support any mechanism of stabilization proposed in the article. The rationality behind ribozyme selection was not well plotted. The ambiguity of some descriptions and misciting the data figure also weaken the work.

The main point of our paper is that molecular crowding, including with prebiotically realistic crowding agents, stimulates ribozymes that carry out RNA synthetic reactions. Furthermore, the stimulation of activity at low concentrations of Mg^{2+} increases the plausibility of ribozyme activity within protocell compartments that are sensitive to Mg^{2+}. Like the reviewer, we would also like to have a full mechanistic understanding of the effects of different crowding agents. However, such experiments are beyond the scope of this paper.

The main criticism from this reviewer is as follows:
To show the beneficial effect of crowding as a general phenomenon, more ribozymes with a larger diversity of sequence should be screened. Or at least all the ribozymes used in the study should be tested with each of the negative affecters that were proposed to be overcome by crowding agents.

While more data is always nice, in this work we have focused on ribozymes that catalyze RNA assembly reactions – RNA ligation and RNA polymerization – as these would have been instrumental in replicating RNA genomes and generating a diversity of RNA enzymes in a primordial RNA-based biology. Ribozymes that catalyze these reactions also generally exhibit high Mg^{2+} requirements,

making them attractive targets for testing the beneficial effects of molecular crowding. In future work we hope to test the effects of molecular crowding on the activities of other ribozymes relevant to the RNA World and the origin of life.

For example, it is questionable why the authors choose not to use ligase 1&2 in the denaturation section. It was stated that s 1 and 2 were inactive at low Mg2+ in the absence of crowding agents. But why did the authors not perform the assay at an intermediate Mg2+ concentration that can show both crowding compensation for Mg2+ and denaturation?

As the reviewer rightly points out, ligases 1 and 2 were not chosen for denaturation experiments as they are inactive at the low [Mg^{2+}] used in that experiment. Therefore, these ribozymes cannot be used to capture the detrimental effects of denaturants or the protective effects of crowding in the presence of denaturants at low [Mg^{2+}]. We decided to use 1 mM Mg^{2+} and ligase 3, a ribozyme that is active at 1 mM Mg^{2+}, in this experiment as the beneficial effects of crowding are the most pronounced at low Mg^{2+}concentrations. Because we are most interested in ribozyme activity at low Mg^{2+} concentrations that are compatible with fatty acid-based protocell membranes, we did not repeat this experiment with other ligases at higher [Mg^{2+}] concentrations. To clarify this point, we modified the text as follows:

"Since crowding promotes the formation of compact RNA folds, we wondered if molecular crowding could protect ribozymes from unfolding under denaturing conditions at the low Mg^{2+} concentrations that are compatible with fatty acid-based protocell membranes. As ligase 1 and ligase 2 are inactive at low Mg^{2+} in the absence of crowding agents, these ribozymes cannot be used to capture the detrimental effects of denaturants or the protective effects of crowding in the presence of denaturants under these low Mg^{2+} conditions. Therefore, we used a previously reported 2AI-ligase (henceforth, ligase 3) that is functional under these conditions for the following experiments.[11]"

It is also unclear if ligase 3 Mg2+ dependence can be rescued by PEG as ligase 1&2.

Ligase 3 has a low Mg^{2+} requirement ($[Mg^{2+}]_{1/2}$ = ~0.9 mM) allowing it to function at 1 mM Mg^{2+} (DasGupta et al. 2023, Chem. Eur. J.) in the absence of molecular crowding. Therefore, this ligase ribozyme is ideal for capturing the reduction in activity under denaturing conditions and the subsequent rescue in ligation upon addition of crowders. Ligase 3-catalyzed ligation reaches its optimal rate at 2 mM Mg^{2+} ,therefore, the effect of molecular crowding on ligase 3 at this Mg^{2+} concentration is minimal.

The minor suggestions from this reviewer are as follows:
1. Fig 1D was not mentioned in the main text.

We thank the reviewer for noticing this error. We now refer to Fig. 1D in the text.

2. PEG 400 has the highest Kobs in Fig.1 but was not discussed specifically.

As we were more interested in the general effect of crowders, we did not compare the beneficial effects of the various PEGs we tested or elaborate on the slightly higher ligation rate in the presence of PEG 400. As mentioned earlier, due to complications in identifying the forces that lead to ribozyme activation in crowded solutions, we prefer to not speculate at this point.

3. Page 6, lines 10-13, the authors proposed an alternative explanation to the induction of RNA folding, but no folding pattern of the ribozyme has been described in this article. They only mentioned activity.

We proposed other possible explanations for completeness, however as noted before detailed mechanistic studies are beyond the scope of this paper.

4. Fig. S3 has no crowder concentration. It is also hard to say if the crowder has no effect because the ligation efficiency of the nonenzymatic ligation is low to start from.

We apologize for the omission. We have now included the concentrations of the crowders in Fig. S3. Any beneficial effect from crowding would be more pronounced when the background activity is low as is the case with nonenzymatic RNA ligation. The fact that there is no detectable increase in non-ribozyme mediated ligation in the presence of crowding suggests that ribozyme structure is important for this effect.

5. Why does S5 use two different [MgCl2] (1 mM in S5A and 2 mM in S5B)?

To maximize the effect of crowders under denaturing conditions, we used the optimal Mg^{2+} concentration in the presence of the particular crowder being used, which is 2 mM for EG and 1 mM for PEG 200 and PEG 1000 (Fig. 2B-D). While we found that PEG 200 and PEG 1000 stimulate ligation under denaturing conditions (Fig. S5A and Fig. 3A), we found no significant benefit under these conditions with EG (Fig. S5B).

6. The term "nucleic acid assembly", especially "assembly", was used in an unclear manner. For example, on Page 8, line 51, the authors wrote "ribozyme-catalyzed RNA assembly" on RNA polymerase activity.

We have used 'RNA assembly' to represent both RNA ligation and RNA polymerization reactions that are discussed in this work. In the sentence the reviewer refers to, we highlighted the fact that both ribozyme-catalyzed RNA ligation and RNA polymerization are stimulated at low [Mg^{2+}] by molecular crowding, suggesting a general beneficial effect of crowding on RNA assembly reactions.

7. Page 8, line 48 should be Fig S8

We thank the reviewer for catching this typo. We have now corrected the error.

Reviewer: 4

Comments:
The manuscript by DasGupta et al. reports molecular crowding-induced stimulation of RNA-catalyzed RNA ligation and polymerization. The authors studied the catalytic activities of several ligase and polymerase ribozymes at low Mg ion concentrations. RNA ligation assays showed enhanced ligation rates and decreased Mg ion requirements for RNA folding in the presence of crowders, EG and PEGs of various MWs. The presence of the crowders protected the ribozyme from unfolding in the presence of high concentrations of urea and in alkaline pH solutions. Prebiotically relevant small molecules such as EG, ribose, and amino acids also facilitated the ligation. The authors propose an important role of molecular crowding in the evolution of RNA-based primordial life. Overall, investigation of the ribozyme activity in crowed environments is very important. This study is carefully designed and the conclusion is reasonable. However, the results of enhanced ribozyme activities in crowed environments are not very surprising (crowding-induced RNA folding and catalysis have already been reported for several types of ribozymes although this study uses ligase and polymerase ribozymes that are relevant to the evolution of earliest living cells). This study appears too specialized to be of interest to the readers of ACS Central Science, and it would be better fit to a more specialized journal.

We respectfully disagree. We submitted this paper to ACS Central Science because of its important implications for ribozyme function during the origin and early evolution of life. The main point of our paper is that molecular crowding, including with prebiotically realistic crowding agents, stimulates ribozymes that carry out RNA synthetic reactions. Furthermore, the stimulation of activity at low concentrations of Mg^{2+} increases the plausibility of ribozyme activity within protocell

compartments that are sensitive to Mg^{2+}. These conclusions are novel and help to answer an important issue in origin of life studies.

I have several other concerns as follows: (1) The mechanisms that stimulate the ribozyme activities in the presence of both large and small molecules are not deeply discussed.

The exact mechanisms by which ribozyme activities are stimulated in the presence of low MW and high MW crowders are not clear for reasons described in our response to Comment 2 by Reviewer 2. However, previous reports with other ribozymes, and observations made in our study such as the insensitivity of nonenzymatic RNA ligation toward crowding and the beneficial effects of crowding under denaturing conditions, suggest that crowding facilitates the formation of folded RNA structures, which increases the fraction of the catalytically relevant fold. More involved experiments directed at investigating the details of how crowders modify the structure and function of ribozymes are beyond the scope of this study.

In particular, how can amino acids stimulate the ligation?

The mechanism by which ribozyme-catalyzed ligation is enhanced by amino acids is not entirely clear. We address this issue in our response to Comment 5 by Reviewer 2. In brief, the observed increase in ligation is likely caused by amino acid-mediated structural compaction of the ligase ribozyme. Localization of Mg^{2+} close to the ribozyme via the formation of a layer of Mg^{2+}-chelating amino acids on the RNA surface may also increase the availability of Mg^{2+} and consequently improve folding and catalysis.

(2) The experiments were conducted at low salt concentrations. However, the amounts of monovalent metal ions such as K and Na ions may not be low in prebiotic environments. RNA base-pairing and folding are strongly influenced by monovalent metal ions when divalent metal ions are absent or very low. Further studies using buffer solutions containing moderate concentrations of monovalent metal ions are needed to draw definite conclusions. Does the authors use salt-fee amino acid solutions? Unless, counter cations of amino acids may affect the efficiency of RNA folding at low Mg ion concentrations. I would like to see the results in the presence of NaCl in place of amino acids, in Figure 4.

We appreciate the opportunity to clarify this point. The ligase ribozyme exhibits negligible ligation at 1 mM Mg^{2+} even in the presence of high concentrations of Na^{+} (300 mM) (Walton et al. 2020, PNAS). Therefore, low concentrations of monovalent counterions in reactions containing 2.5 mM - 20 mM amino acids are unlikely to cause the pronounced rate stimulation we observed. Additionally, ligation rates are not affected by changing [Na^{+}] at optimal [Mg^{2+}] (DasGupta et al., 2023, Chem.

Eur. J). The most likely explanation is that amino acids directly activate ribozyme function at low [Mg^{2+}] by mechanisms discussed above.

We now include the following sentence to clarify this point:

"As ligase 1 exhibits negligible ligation at 1 mM Mg^{2+} even in the presence of high concentrations of Na^+ (300 mM),[6] low concentrations of monovalent counterions in reactions containing 2.5 mM - 20 mM amino acids are unlikely to cause this pronounced rate stimulation and the amino acids must be playing a direct role."

(3) Based on the ligation yields, I suppose the reaction does not complete (the fraction of active complex is not close to 1.0). Are the kinetic data for RNA-catalyzed reactions fit to a single exponential function without a burst phase?

These ribozyme-catalyzed ligation reactions do not go to completion, most likely due to the presence of a fraction of misfolded ribozymes in the total population. This is accounted for in the modified pseudo-first order rate equation used to fit the kinetics data: $y = A(1 - e^{-kx})$, where A represents the fraction of active complex, k is the first order rate constant, x is time, and y is the fraction of ligated product. The substrate is in 2-fold excess solely to ensure that every active ribozyme/template complex has a bound substrate. Given the extent of the base-pairing, the reaction is expected to be a pseudo-first order with respect to the unreacted ribozyme (or ligated product). This interpretation is consistent with our observations.

(4) UV melting experiments were conducted with the ligase ribozyme. However, the complex formation between the ribozyme, substrate, and template is crucial for the preservation of ribozyme activity. It would be best to measure the thermal stability of the RNA complex.

We agree that association of the ribozyme with the substrate and template is essential for its function. However, the purpose of UV melt experiments was to investigate the effect of crowding on thermal stabilization of the folded ribozyme structure. Since the ribozyme-substrate-template ternary complex consists of ribozyme-substrate and substrate-template duplexes, melting of these duplexes leading to complex dissociation, in addition to the melting of the ribozyme secondary structure, would complicate data interpretation.

(5) It is better to mention the quantitativity of RNA stain using SYBR Gold. The extent of fluorescence enhancement upon SYBR Gold binding to RNA is not the same between the unligated precursor and ligated product because of different RNA lengths.

Intensities corresponding to the ligated product were normalized to account for the difference in size between the 95 nt precursor band and the 111 nt product band. SYBR Gold-stained RNA bands can be used to accurately measure kinetics as described in detail by Guillen et al. (PLoS One, 2020).

(6) On page 5, line 11: the amount of PEG400 is missing, which is probably 30%.

We thank the reviewer for pointing this out. We have corrected the omission.

(7) The reaction temperature for ligation assays is not stated. Concentrations of ribose and amino acids for the data of Figure 4 are not described.

All ligation reactions were performed at room temperature, except for experiments assaying for temperature dependence, which were performed at 55 °C. We have now mentioned this in the Experimental Procedures section. Also, we have now mentioned the concentrations of ribose and the various amino acids in Fig. 4.

oc-2023-00547w.R2

Name: Peer Review Information for "Molecular crowding facilitates ribozyme-catalyzed RNA assembly"

Second Round of Reviewer Comments

Reviewer: 1

Comments to the Author

The authors have dealt with all the technical points satisfactorily. The relevance to prebiotic chemistry is high as the crowding phenomenon suggests a means of overcoming a major barrier to RNA replication. In contrast, in extant systems crowding is signicant, but not necessarily a game changer. My feeling is that the paper now merits publication without further changes.

Author's Response to Peer Review Comments:

Dear Dr. Editor,

We appreciate the quick response and the acceptance of our manuscript.

We have addressed the formatting issue mentioned in your letter and attached a 'clean' copy of our manuscript that reflects the change.

Sincerely,

Jack Szostak
